# M. Claude Bernard

**Published:** 1861-05

**Authors:** 


M. CLAUDE BERNARD has recently been elected a member of the Academy of Medicine of Paris, by seventy-two out of seventy-nine votes, in the section of anatomy and physiology. The other candidates were MM. Béclard, Sappey, Verneuil, and Bérand.

